# Agonistic display or courtship behavior? A review of contests over mating opportunity in butterflies

**DOI:** 10.1007/s10164-016-0487-3

**Published:** 2016-09-05

**Authors:** Tsuyoshi Takeuchi

**Affiliations:** Entomological Laboratory, Graduate School of Life and Environmental Science, Osaka Prefecture University, Gakuencho1-1, Nakaku, Sakai, 5998531 Japan

**Keywords:** Competition, Compound eyes, Erroneous courtship, Lloyd Morgan’s Canon, Mating success, Pupal mating, Sexual recognition, Territory

## Abstract

Male butterflies compete over mating opportunities. Two types of contest behavior are reported. Males of various butterfly species compete over a mating territory via aerial interactions until one of the two contestants retreats. Males of other butterfly species fly around larval food plants to find receptive females. Males of some species among the latter type can find a conspecific pupa, and they gather around it without expelling their rivals. Scramble competition over mating occurs when a female emerges from the pupa. Many studies have been performed on territorial species, and their contest resolution has often been understood from the point of view of contest models based on game theory. However, these models cannot explain why these butterflies perform contest displays despite the fact that they do not have the ability to attack their opponent. A recent study based on Lloyd Morgan’s Canon showed that territorial contests of male butterflies are better understood as erroneous courtship between sexually active males. In this paper, I review research on contests over mating opportunity in butterflies, and show that the erroneous courtship framework can explain not only territorial contests of butterflies but also why males do not determine the owner of a conspecific pupa.

## Introduction

Animals compete over limited resources in nature such as mates, food or shelter (reviewed by Hardy and Briffa 2013). Physical attack sometimes occurs, attended by the risk of serious injury or death. The outcome of such fights is often settled on the basis of asymmetry in resource holding potential (RHP) (sensu Parker 1974). In theoretical terms, individuals with higher RHP are able to inflict greater costs on their opponent and minimize their own cost accrual. RHP is usually correlated with body size or weaponry (reviewed by Arnott and Elwood 2009). Game theory has played a central role in constructing frameworks to understand animal contests since the landmark paper of Maynard Smith and Price (1973).

Although RHP is usually correlated with morphological structures, not all animals that compete exhibit such morphological adaptations. Butterflies provide the most typical example of this because they do not have weapons or organs such as teeth, nails or horns with which to injure their opponents. Butterflies rarely compete over food or shelter (but see Peixoto et al. 2012). However, male butterflies compete over mating opportunity, yet lack any obvious means to impose costs on their opponent. Two types of contest behavior are well known. Males of various butterfly species perch on twigs, leaves or the ground in their mating territory, which is located at a sunspot or a small open space in forests, etc., and compete over the territory via aerial interactions (reviewed by Kemp and Wiklund 2001). In many cases, these aerial interactions are non-contact, which makes it difficult to estimate costs imposed on the contestants. On the other hand, not all butterflies have a territorial mating system. Males of other butterfly species perform patrolling: flying around larval food plants to find receptive females (Rutowski 1991). Since a first comer can mate with a female in this mating system, males do not necessarily compete directly over a female. However, in certain cases, males directly compete over mating opportunity. Males of some butterfly species can find conspecific pupae that are going to emerge within a few days. Sometimes several males gather around a pupa, and scramble competition occurs among them when a female emerges (e.g., Elgar and Pierce 1988; Deinert et al. 1994; Deinert 2003).

In addition to the apparent lack of ability to inflict costs on their opponent, further difficulties are imposed by the limitation of butterflies’ cognitive abilities. That is, it is not clear that butterflies are able to recognize whether their opponent is a rival during male–male aerial interactions (Suzuki 1976), although usual contest models assume that contestants can distinguish between rivals and others such as potential partners (e.g., Maynard Smith and Price 1973; Enquist and Leimar 1983; Mesterton-Gibbons et al. 1996; Payne 1998; but see Yabuta 2008). Reflecting these difficulties, the issue of contest resolution in butterflies has been controversial (Baker 1972; Suzuki 1976; Davies 1978; Austad et al. 1979; Stutt and Willmer 1998; Hardy 1998; Field and Hardy 2000; Kemp 2000a, 2013; Kemp and Wiklund 2001, 2004; Takeuchi et al. 2016). The persistence of such a long-lasting controversy means that behavioral ecologists have remained interested in this issue. Butterflies provide an ideal opportunity to examine the evolution of a contest system that is not simply influenced by the ability to attack rivals.

In this paper, I review research on contests over mating opportunity in butterflies. First, I give an overview of the theoretical basis for understanding animal contests. Second, I review the relevant empirical studies on butterflies, namely, studies on contests over a mating territory, and on contests over a conspecific pupa. Last, I try to understand the two forms of contests comprehensively, and propose future directions for research on this issue.

## Theoretical models

First, a “contest” should be defined. I basically adopt the definition of a contest used by Hardy and Briffa (2013), namely, a direct and indirect behavioral interaction that determines the ownership of an indivisible resource unit. In this paper, however, a contest can include a behavioral interaction over an indivisible resource unit that fails to determine the owner.

Three contest models have been subjects of intensive empirical research in recent years because these models can provide testable predictions (Arnott and Elwood 2009; Hardy and Briffa 2013). These models are based on game theory, which deals with situations where two or more individuals conflict, and finds equilibrium solutions termed evolutionary stable strategies. The sequential assessment model assumes that contest behavior is a sampling of the opponent’s RHP, and that the individual that recognizes that its RHP is lower than its opponent’s should retreat in order to avoid contests that it would inevitably lose (Enquist and Leimar 1983). The cumulative assessment model assumes that a contestant retreats when accumulated costs from both its own and its opponent’s actions reach their threshold (Payne 1998). The war of attrition model assumes that a contestant retreats when accumulated costs from its own actions reach their threshold (Maynard Smith and Price 1973; Mesterton-Gibbons et al. 1996). Recently, Mesterton-Gibbons and Heap (2014) tried to understand these models as evolution from self assessment (wars of attrition and cumulative assessment) to mutual assessment (sequential assessment). The three models have different assumptions about the functions of agonistic behavior, and provide different predictions concerning contest duration and/or dynamics (Taylor and Elwood 2003; Arnott and Elwood 2009). I summarize the assumptions and predictions in Table 1. There have been many studies investigating which of the predictions of the three models best explain the contest dynamics of target animals (reviewed by Hardy and Briffa 2013).

**Table 1:** Key features of the three major contest models

Model	Retreating decision based on	The relationship between contest duration and resource holding potential (RHP)	Contest dynamics and structure
Sequential assessment model (Enquist and Leimar 1983)	Information that the opponent has greater RHP	Loser (+) Winner (−)	Progressing into increasingly intense phases, but constant within phases
Cumulative assessment model (Payne 1998)	A threshold cost that is a result of the loser’s own and the opponent’s actions	Loser (+) Winner (−)	Constant, escalating or de-escalating during contests
War of attrition model (Mesterton-Gibbons et al. 1996)	A threshold cost that is a result of the loser’s actions	Loser (+) Winner (0 or +)	Constant, escalating or de-escalating during contests

Kemp and Wiklund (2001) claimed that territorial contests of butterflies are excellent examples of the war of attrition family of theoretical models that deal with contests (1) that consist only of display, (2) where costs are inflicted continuously, (3) the outcome of which is determined by persistence. Since then, territorial contests of butterflies have frequently been regarded as wars of attrition (Kemp 2002a, 2003, 2013; Kemp and Wiklund 2004; Takeuchi 2006a, b, 2011; Takeuchi and Honda 2009; Bergman et al. 2010). There have also been a few studies suggesting that contest dynamics of butterflies accord with a sequential assessment model (see Table 1) (Kemp 2000b, 2003).

One potential pitfall of such a hypothesis-testing approach is that the same prediction can be drawn from other models that are based on premises different from those of the three contest models. In such cases, researchers cannot distinguish among the three models and the other models by analyzing which of the predictions of the three models best explain the data obtained about contest dynamics. If the animals’ characteristics do not fulfill the premises of the candidate models, it is meaningless to claim that obtained data fit the prediction of the models. Therefore, in empirical research, testing the premises of the candidate models is as important as testing the predictions of the models (Kokko 2013). All three contest models assume that (1) animals can inflict costs on their opponent even if the opponent does not exhibit contest behavior such as display, and that (2) animals can discriminate their rivals from other individuals such as partners or natural enemies during contests. The first of these premises is required because if it is not fulfilled, cheats who delay or stop their display to gain an energy advantage can invade (Payne 1998). Clearly these two premises have not been confirmed for every animal yet.

Takeuchi et al. (2016) critically reviewed past research on territorial contests of butterflies, and found no evidence that the two premises were fulfilled. Takeuchi et al. (2016) then presented another framework to understand butterfly contests, termed the erroneous courtship hypothesis, that does not require the two premises. The erroneous courtship hypothesis is not based on game theory in that it does not analyze the evolution of strategies. The logical basis of the hypothesis is the principle of parsimony in comparative psychology, i.e., Lloyd Morgan’s Canon: in no case is an animal’s activity to be interpreted in terms of higher psychological processes if it can be fairly interpreted in terms of processes which stand lower on the scale of psychological evolution and development (Morgan 1894). The erroneous courtship hypothesis is based on two premises: butterflies are not able to inflict significant costs on a non-displaying opponent, and butterflies are not able to fully discriminate the sex of flying conspecifics. Under these premises, aerial contests of territorial butterflies are viewed as follows. Males chase each other expecting that their opponent is a receptive female. One of the two males retreats when the probability that his opponent is a receptive female falls below the level acceptable to him. Assuming that this acceptable level is determined by the abilities and/or experiences of individuals, the erroneous courtship hypothesis can also explain the contest duration predicted by the three major contest models.

## Contests over mating territory

Males of many butterfly species compete over mating territories. In these contests, butterflies of the various species perform similar aerial interactions (Kemp and Wiklund 2001; Kemp 2013). A territorial male occupies a specific area, such as a sunspot in the woods or a lookout point, where there are no obvious resources such as food or oviposition sites, but to which females sometimes come. When a female flies into the territory, the owner detects her visually (Rutowski 1991), and chases her, which sometimes results in copulation. During this process, the pair does not copulate in the air. If a female flying into the territory alights nearby, the male also alights there, walks to her, and bends his abdomen to copulate with her (e.g., Wickman and Wiklund 1983; Takeuchi and Imafuku 2005; Takeuchi 2010). When another male intrudes into the area, the territorial male takes off towards him. Then, the two males fly around each other, followed by a horizontal chase without apparent physical attack until one of them leaves the area. Therefore, their aerial interactions function as contests because an owner of the territory is determined via this behavior. In a few species, males perform horizontal chases for a long time (Takeuchi 2011). Research performed before 2000 was reviewed by Kemp and Wiklund (2001). Here, I will mainly focus on research performed since then.

The first question is whether possessing a territory actually increases the mating success of its owner. Surprisingly, there are only a handful of studies investigating the reproductive advantages of territory owners in butterflies. Perhaps this is because mating of territorial butterflies is rarely observed (e.g., Cordero 2000; Kemp 2000b; Takeuchi and Imafuku 2005). In *Lycaena hippothoe*, matings were more frequently observed within territories than outside of territories (Fischer and Fiedler 2001). Similar results were obtained in *Lethe diana* (Takeuchi 2010). Bergman et al. (2007) performed a cage experiment that revealed that males of the speckled wood butterfly, *Pararge aegeria*, occupying sunspot territories enjoyed higher mating success than males wandering in the shade. Although more studies are required, these findings showed that occupying territories enhances the mating success of owners.

The second question is how contests of territorial butterflies are resolved. From the point of view of the usual contest models, the particular difficulty in butterfly contests is that it is unclear what kind of costs they can impose on the opponents. Kemp and Wiklund (2001) pointed out this difficulty at the beginning of the twenty-first century; however, no one has determined the actual costs in butterfly contests yet. The territorial contest of butterflies came to be considered a good example of the bourgeois strategy [to escalate in a resident role, and to retreat in an intruder role (Maynard Smith and Parker 1976)], when Davies (1978) showed in *P. aegeria* that owners temporarily removed from their territory could not retake the territory after it became occupied by a newcomer. Presumably the bourgeois strategy was accepted because there seemed to be no other explanation of how these weaponless animals could otherwise settle their contests. However, later studies revealed the opposite results: in *P. aegeria* an owner of territory that leaves it temporarily can retake the territory when it has been occupied by a newcomer (Wickman and Wiklund 1983; Kemp and Wiklund 2004). Although the bourgeois strategy has also been disproved in other species (Takeuchi 2006a; Peixoto and Benson 2012), a type of residency effect exists in butterfly territorial systems as well as in other animals (Sherratt and Mesterton-Gibbons 2015). In the hairstreak *Chrysozephyrus smaragdinus*, males that have occupied the contested territory longer tend to chase their opponent longer, and as a result, tend to win irrespective of their morphological or physiological characteristics (Takeuchi 2006a, b, 2016; Takeuchi and Honda 2009). Similar results were reported in *Melanitis leda* (Kemp 2003) and *P. aegeria* (Kemp and Wiklund 2004). Takeuchi et al. (2016) pointed out that these results can be interpreted in the context of the erroneous courtship as follows: males reduce the level of risks (such as predation risk) arising from continuing aerial interaction, and consequently chase their opponent longer as their residence duration increases.

Although fighting costs in butterflies are unclear, differences in morphological or physiological characteristics between territory owners and intruders are found in many species (Table 2). For example, owners are larger than intruders in some butterflies (Rosenberg and Enquist 1991; Martínez-Lendech et al. 2007; Peixoto and Benson 2008, 2011, 2012; Takeuchi 2011; Carvalho et al. 2016). The effect of large body size on winning contests is ubiquitous in the animal kingdom (Hardy and Briffa 2013). However, owners are smaller than intruders in *Heliconius sara* (Hernandez and Benson 1998). Differences in body size between owners and intruders were not found in other specie (Lederhouse 1982; Kemp 2000b, 2003; Takeuchi 2006a; Kemp and Wiklund 2004; Bergman et al. 2007; Da et al. 2016). Owners have a large flight muscle ratio in *Lethe diana*, suggesting that acceleration and/or maneuverability is important (Takeuchi 2011). In contrast, owners have smaller flight muscle ratio in *Hermeuptychia fallax* (Peixoto and Benson 2012). Owners have large fat reserves in *H.* *fallax* (Peixoto and Benson 2011), suggesting that their contests are energetic wars of attrition. In contrast, owners have smaller fat reserves in *C. smaragdinus* (Takeuchi 2006b). Owners are younger in *Melanitis leda* (Kemp 2003). In contrast, owners are older than intruders in *Hypolimnas bolina* (Kemp 2000b) and *P. aegeria* (Kemp et al. 2006a) although body condition declines during the course of adult life (Kemp 2002b). These results were interpreted as indicating that older males invest more in a present reproductive chance because they have fewer future reproductive chances. Although various butterfly species exhibit similar forms of aerial contests, characteristics that are correlated to ownership vary widely among species.

**Table 2:** Relationship between characteristics and territorial status

Characteristics	Relationship to territorial status
Body size	+: *Limenitis weidemeyerii* (Rosenberg and Enquist 1991), *Eumaeus toxea* (Martinez-Lendech et al. 2007), *Paryphthimoides phronius* (Peixoto and Benson 2008), *Lethe diana* (Takeuchi 2011), *Moneuptychia soter* (Peixoto and Benson 2011), *Hermeuptychia fallax* (Peixoto and Benson 2012), *Actinote pellenea* (Carvalho et al. 2016) −: *Heliconius sara* (Hernandez and Benson 1998) 0: *Papilio polyxenes* (Lederhouse 1982), *Hypolimnas bolina* (Kemp 2000b); *Melanitis leda* (Kemp 2003), *Pararge aegeria* (Kemp and Wiklund 2004; Kemp et al. 2006a; Bergman et al. 2007), *Chrysozephyrus smaragdinus* (Takeuchi 2006a,b), *Hermeuptychia fallax* (Peixoto and Benson 2011), *Parnassius imperator* (Da et al. 2016)
Flight muscle ratio	+: *Lethe diana* (Takeuchi 2011) −: *Hermeuptychia fallax* (Peixoto and Benson 2012) 0: *Hypolimnas bolina* (Kemp 2002b), *Chrysozephyrus smaragdinus* (Takeuchi 2006b), *Pararge aegeria* (Kemp et al. 2006a), *Hermeuptychia fallax*, *Moneuptychia soter* (Peixoto and Benson 2011)
Fat reserve	+: *Hermeuptychia fallax* (Peixoto and Benson 2011) −: *Chrysozephyrus smaragdinus* (Takeuchi 2006b) 0: *Hypolimnas bolina* (Kemp 2002b), *Eumaeus toxea* (Martinez-Lendech et al. 2007), *Lethe diana* (Takeuchi 2011), *Moneuptychia soter* (Peixoto and Benson 2011)
Age (wing wear)	+: *Hypolimnas bolina* (Kemp 2000b), *Pararge aegeria* (Kemp et al. 2006a) −: *Melanitis leda* (Kemp 2003), *Hermeuptychia fallax* (Peixoto and Benson 2011) 0: *Chrysozephyrus smaragdinus* (Takeuchi 2006b), *Paryphthimoides phronius* (Peixoto and Benson 2008), *Lethe diana* (Takeuchi 2011), *Moneuptychia soter* (Peixoto and Benson 2011)

*Plus symbol* Winners (owners) have higher (older) value,* minus symbol* winners (owners) have smaller (younger) value,* zero* no significant difference was detected between winners (owners) and losers (intruders)

Characters that differ between owners and intruders have often been regarded as RHP-correlated characters, and researchers have often interpreted owners’ and intruders’ behavior based on the three major contest models by analyzing contest dynamics (Kemp 2000b, 2003; Kemp et al. 2006a; Peixoto and Benson 2012). Of the three contest models, the predictions of sequential assessment or the war of attrition best fit the contest dynamics of these butterflies. However, Takeuchi et al. (2016) pointed out that the erroneous courtship hypothesis can also explain these results, considering that the butterflies’ species recognition is uncertain and there is a possibility that an opponent is a natural enemy. That is, a male with an inferior ability (or lower motivation) should retreat earlier.

In some species, aerial clashes occur. These cases have been reported as “rare examples” of butterfly contests. Carvalho et al. (2016) reported that males of the Neotropical butterfly *Actinote pellenea* grab their opponent in the air and fall to the ground in 10 % of their territorial contests. Males with larger body size tend to win the contests. Their contests can be interpreted as erroneous courtship because also in male–female aerial interactions, a male grabs a female, causing both to fall to the ground. However, males might be able to impose physical costs on their opponent by an erroneous copulation attempt, and larger males would impose more costs on their opponent than smaller males. In such cases, usual contest models may be applicable. Lehnert et al. (2013) reported that males of the Homerus swallowtail, *Papilio homerus*, clash during their aerial interactions. Unfortunately, the form of male–female interactions of *P. homerus* is unknown, perhaps because this species is rare (Garraway et al. 2008). Chaves et al. (2006) reported the unusual case of the lekking butterfly *Charis cadytis*: after aerial interactions, two males sometimes alight on a leaf, and push each other with their wings. However, they are not injured by the contact, and it is normally not possible to determine which male wins the pushing contest because they often restart aerial interactions after the pushing phase. In *C. cadytis*, two male–female interactions were observed: in both cases, a male flew after a passing female, which immediately landed and mated without further ado (Chaves et al. 2006). Because the two females were receptive and they did not resist the male, the degree of similarity between male–male and male–female interactions is not yet sufficiently known in this species.

## Contest over conspecific pupae

Not all butterfly species have a mating territory. Males of some butterfly species instead fly around larval food plants to find receptive females (Rutowski 1991). Among such species, there are some in which males aggregate around a conspecific pupa that is close to emergence, and copulate with a female when she emerges (Watanabe 1978; Fukuda et al. 1982, 1983; Elgar and Pierce 1988; Kato and Nakane 1989; Deinert et al. 1994; Hernandez and Benson 1998; Deinert 2003; Estrada et al. 2010; Walters and Harrison 2011). Whether males can discriminate the sexes of pupae depends on the species. *Heliconius hewitsoni* (Deinert 2003) and *Heliconius charithonia* (Estrada et al. 2010) can distinguish the sexes of pupae, whereas *Jalmenus evagoras* (Elgar and Pierce 1988) and *Eurema hecabe* (Kato and Nakane 1989) cannot.

Quantitative studies on this type of contest were made on two species, *H. hewitsoni* (Deinert et al. 1994; Deinert 2003), and *J. evagoras* (Elgar and Pierce 1988). The pupal mating system of *H. hewitsoni* has been well studied. Gathering males sit around a female pupa. Larger males enjoy advantages in sitting on a pupa. However, they do not necessarily remove the rivals. That is, they do not determine an owner of the pupa (an indivisible resource). When a female emerges, gathering males try to grasp her and to copulate. Interestingly, smaller males enjoy advantages at this stage. Consequently, there is no net advantage dependent on body size in *H. hewitsoni*. The mating system of *J. evagoras* provides another case. Males aggregate around conspecific pupae without discriminating the sexes of the pupae. Not only a pupa itself but also the presence of conspecific males is used as a cue to find pupae. Males do not determine an owner of a pupa, and therefore sitting males form a “mating ball” on the pupa. Scramble competition occurs when a female emerges. Larger males enjoy higher mating success in this species.

## Clinging behavior

Suzuki and Matsumoto (1990) reported an unusual form of mate competition in butterflies. Males of the papilionid butterfly *Atrophaneura alcinous* fly around larval food plants to find receptive females. When males find a conspecific mating pair, they cling to the pair, and clinging males copulate with the female after the previous copulation ends. Up to five males were observed clinging to a mating pair. Although males make a mating plug at the ostium bursae of their sexual partner, about 50 % of remating males succeeded in transferring a spermatophore to the female because mating plugs were still soft at this time. The male that had mated with the female previously did not expel the clinging males. At present, clinging behavior has not been reported in other butterfly species.

## Discussion

In butterflies, there is a clear difference in the style of contests between territorial males and males that gather around pupae. In contests over territories, one male monopolizes a territory (a future mating chance), whereas in a pupal mating system, two or more males do not determine an owner of a pupa (a future mating chance). The fact that scramble competition over an emerging female occurs indicates that the owner of a pupa was not determined. Why do males not determine the owner of a pupa, a clearly indivisible resource, although males determine the ownership of a mating territory? If males can expel their rivals by aerial interactions, not only territorial males but also males gathering around conspecific pupae should compete via aerial interactions. It is difficult to explain why there is such a difference using the framework of the usual contest models. One might think that it is due to a difference between aggressive species and gentle species. However, the mating system of *H. sara* rules out this possible explanation because males of this species exhibit both territorial and pupal mating systems (Hernandez and Benson 1998).

**Fig. 1:**
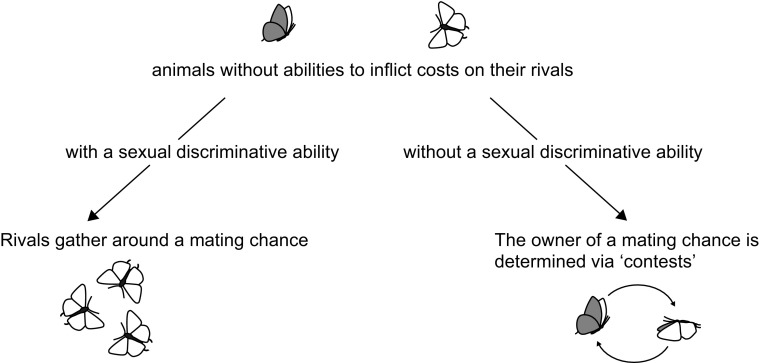
The forms of mate competition according to the logic of the erroneous courtship hypothesis

Here I apply the erroneous courtship framework to answer this question. This framework posits that the possibility of the opponent being a receptive female invokes “contests” of butterflies that are not able to inflict costs on their opponent. Consequently, contests would not occur when neither of the males expects that its opponent is a receptive female. Males sitting around a conspecific pupa do not perform mating behavior towards each other, indicating that they do not confuse the sexes. In fact, males of *H. charithonia* find pupae using a volatile pheromone as a key stimulus (Estrada and Gilbert 2010; Estrada et al. 2010). Therefore, in contrast to territorial males, aerial contests should not occur between males gathering around pupae (Fig. 1).

Males of *A. alcinous* allow another male to copulate with a female after their own copulation with her. This suggests that the butterfly cannot inflict costs on its rivals, and therefore cannot physically expel them. This is congruent with the premises of the erroneous courtship hypothesis. In contrast, odonates that can attack their opponent exhibit mate-guarding behavior after copulation (Corbet 1999). Butterflies have evolved extraordinarily elaborate mating plugs (Simmons 2014). This may be because male butterflies cannot expel their rivals by physical attack, and instead invest their resources in a mating plug to ensure paternity.

One characteristic property of territorial contests of butterflies is that morphological or physiological traits correlated with contest success vary widely among species. Moreover, the relationship between traits and contest success was inconsistent between studies in *H. fallax* (Table 2). This contrasts with contests of other animals, where body size is usually correlated with RHP (Arnott and Elwood 2009). If contestants can impose physical costs on their opponent, the contestants with larger body size are generally expected to enjoy advantages because they can generate larger kinetic energy. The fact that traits correlated with contest success vary widely among species suggests that the ability to inflict physical costs on their opponent plays a minor role in butterfly contests.

It should be noted that the erroneous courtship hypothesis is based on a null hypothesis that males cannot fully discriminate the sexes of flying conspecifics. Consequently, the premise of the erroneous courtship hypothesis cannot be directly tested. Based on Lloyd Morgan’s Canon, the erroneous courtship hypothesis should be adopted as long as there is no evidence that males can fully discriminate the sex of flying conspecifics. If one could find such evidence, for instance, of territorial males rushing towards intruding males and attacking them, but not rushing towards intruding females but rather exhibiting courtship display towards them in their territories, one could rule out the erroneous courtship hypothesis. Therefore, not only the form of male–male interactions, but also that of male–female interactions, is important for understanding aerial contests of butterflies.

### Future directions

At present, the erroneous courtship hypothesis is adequate for butterfly contests, since it is based on simpler cognitive assumptions than the usual contest models, and does not contradict the inability of butterflies to impose costs on their opponent. However, this is based on the available evidence, which is insufficient. Butterflies depend largely on vision for their communication, and strong volatile female pheromones like those of moths have not been reported in this taxon (Rutowski 1991; Vane-Wright and Boppré 1993; Sarto i Monteys et al. 2016). However, butterflies use sex pheromones at a close distance (Sarto i Monteys et al. 2016). Males engaging in an aerial interaction might evaluate the sex of their opponent using odor. In any case, a key factor in understanding contest behavior in butterflies is sex discriminative abilities. If one tries to apply the usual contest models to territorial contests of butterflies, the ability to recognize sexes of flying conspecifics must first be tested.

It is relatively easy to test the color vision of animals because one can use learning interrogation for this purpose (Marshall and Arikawa 2014). In contrast, it is much more difficult to examine the spatial acuity of animals, which is important for recognizing the identity of objects. Spatial acuity of compound eyes has often been inferred from the results of measuring interommatidial angles on the basis that smaller interommatidial angles enable higher spatial acuity (Land 1997). Past research indicated low spatial resolution of butterfly compound eyes (Rutowski and Warrant 2002; Rutowski et al. 2009). To measure interommatidial angles, live animals and a goniometer are required, which makes the measurement rather laborious. Recently, Bergman and Rutowski (2016) have developed a convenient method to measure interommatidial angles of compound eyes using micrographs of dead animals and focus stacking. This method will help to evaluate the spatial acuity of butterfly eyes.

However, we should remember that this is a crude estimate of animal vision. Real cognitive abilities must be tested by behavioral experiments. Unfortunately, most territorial butterflies do not respond to static models, which makes it difficult to study their cognitive abilities (e.g., Warzecha and Egelhaaf 1995). Recently, Imafuku and Kitamura (2015) developed rotating wing models using a motor, and succeeded in inducing responses of territorial males of two hairstreaks. Although their experimental results should be interpreted carefully (Takeuchi 2015), behavioral experiments using such devices will provide information on the cognitive abilities of butterflies required for testing the premise of the usual contest models.

From the point of view of erroneous courtship, the reason why butterfly aerial interactions end is a problem. Takeuchi et al. (2016) proposed the erroneous courtship model assuming that a male–male territorial interaction ends when one male judges that the probability that its opponent is a receptive female is smaller than the threshold level based on the flight duration of the opponent. However, it is also likely that a male evaluates the probability that its opponent is a female based on the shape, movement or odor of its opponent, and that the duration of their interactions reflects the time required to gather information on the identity of the opponent (however, uncertainty should remain because if a male can know that its opponent is not a natural enemy but a conspecific male, there is no reason why one of the males would leave the territory). Territorial males chase not only conspecifics but also heterospecifics such as other butterfly species, other insects, birds or thrown boards (e.g., Tinbergen et al. 1972; Lederhouse et al. 1992; Bergman and Rutowski 2015). By analyzing the form and duration of the interactions with various objects, one may reveal which assumption about cognitive ability is better.

Cognitive ability is the key to the more general question of why some species of butterflies have a territorial mating system while others do not. Bergman et al. (2007) showed that males of *P. aegeria* can detect passing females more frequently in their territories (sunspots) than in the shade, although the frequency of female visits did not differ within compared to outside their territories. This result suggests that territories are sites where male butterflies can minimize the disadvantages of their low cognitive abilities. Possibly, butterflies that have difficulty in searching for mates have a territorial mating system.

As mentioned above, studies investigating whether territories really provide mating advantages to owners are very limited in butterflies, perhaps because counting the number of matings that each male achieves is difficult in nature. Recently, Sasaki et al. (2015) developed a method to infer recent mating experiences of male butterflies. They found that the simplex (a part of the male reproductive organ) is shortened for 2 days after copulation in the pipevine swallowtail butterfly, *Battus philenor*. Such methods open the way to study individual mating success of butterflies in nature. However, there is a cautionary point. Territory owners of the hairstreak *Chrysozephyrus smaragdinus* disappeared from their territories after copulation (Takeuchi and Imafuku 2005). Perhaps mated males had a worsened body condition, since male butterflies transfer a large amount of ejaculate to their sexual partner (e.g., Svärd and Wiklund 1989; Stjernholm and Karlsson 2006; Takeuchi 2012). That is, the mating experience may change their mating strategy. Thus, the present mating strategy of an individual may not represent its mating strategy when a recent mating was performed.

Some other flying insects, such as moths (Sarto i Monteys et al. 2016), odonates (Corbet 1999), wasps and flies (e.g., Kemp and Alcock 2003), also exhibit aerial territorial contests. In the territorial wasp *Hemipepsis ustulata*, physical injury during aerial contests was never observed during 20 years of study (Kemp et al. 2006b). The erroneous courtship hypothesis may provide new insights into the contest behavior of these animals.
